# Indicators, spatial distribution, and hidden endemicity of Hansen’s disease: descriptive study, Piauí, 2018–2022

**DOI:** 10.1590/S2237-96222026v35e20250504.en

**Published:** 2026-07-20

**Authors:** Khaab Gibran Leal Vasconcelos, Lara Beatriz de Sousa Araújo, Raimundo Leoberto Torres de Sousa, Olívia Dias de Araújo

**Affiliations:** 1 Universidade Federal do Piauí Teresina Teresina PI Brazil Universidade Federal do Piauí, Teresina, PI, Brazil; 2 Universidade Federal do Piauí Programa de Pós-Graduação em Saúde e Comunidade Teresina PI Brazil Universidade Federal do Piauí, Programa de Pós-Graduação em Saúde e Comunidade, Teresina, PI, Brazil; 3 Centro de Inteligência em Agravos Tropicais Emergentes e Negligenciados Teresina Teresina PI Brazil Centro de Inteligência em Agravos Tropicais Emergentes e Negligenciados, Teresina, PI, Brazil

**Keywords:** Leprosy, Health Status Indicators, Indicators of Health Services, Public Health, Epidemiologic Studies, Lepra, Indicadores de Salud, Indicadores de Servicios, Salud Pública, Estudios Epidemiológicos

## Abstract

**Objective:**

To analyze epidemiological and operational indicators and the hidden endemicity of Hansen’s disease in Piauí from 2018 to 2022.

**Methods:**

A descriptive study with spatial analysis, using data provided by the State Health Department of Piauí. The variables analyzed included the detection rate of new cases in individuals under 15 years of age, the proportion of new cases presenting grade 2 physical disability at diagnosis, and the percentage of contacts of new cases who were evaluated. Indicators were presented as absolute frequencies per municipality, with percentage variation calculated. Thematic maps were created, and the hidden prevalence of Hansen’s disease in the state was estimated.

**Results:**

A total of 3,789 new cases were identified. Based on hidden prevalence, an estimated 1,265 cases went undiagnosed during the period. In 2018, 27 municipalities were considered hyperendemic regarding detection in individuals under 15 years, decreasing to 11 by the end of the series. The distribution of new cases with grade 2 disability at diagnosis was predominantly classified as “low” throughout the period, ranging from 143 to 178 municipalities. The proportion of contacts examined for new cases in the “poor” status increased the number of municipalities by 28.8% during the period.

**Conclusion:**

Hansen’s disease remains hyperendemic in the state, with high detection rates in individuals under 15 years, a substantial proportion of cases with grade 2 physical disability at diagnosis, and weaknesses in surveillance and control actions. The high estimated number of hidden cases underscores the need for strategies to expand early detection and strengthen Primary Health Care.

## Resumen

**Objetivo:**

Analizar los indicadores epidemiológicos y operativos, así como la endemia oculta de la lepra en Piauí, en el período de 2018 a 2022.

**Métodos:**

Estudio descriptivo, con análisis espacial, utilizando datos proporcionados por la Secretaría de Estado de Salud de Piauí. Las variables analizadas fueron: tasa de detección de casos nuevos en menores de 15 años; proporción de casos nuevos con grado 2 de discapacidad física al momento del diagnóstico; y porcentaje de evaluación de contactos de los nuevos diagnósticos. Los indicadores se presentaron en frecuencia absoluta por municipio, con cálculo de la variación porcentual. Se elaboraron mapas temáticos y se estimó la prevalencia oculta de la lepra en el estado.

**Resultados:**

Se identificaron 3.789 casos nuevos. Según la prevalencia oculta, se estima que 1.265 casos no fueron diagnosticados durante el período. En 2018, 27 municipios fueron considerados hiperendémicos en la detección de menores de 15 años, reduciéndose a 11 al final de la serie. La distribución de casos nuevos con grado 2 de discapacidad al diagnóstico se presentó mayoritariamente en el parámetro “bajo” durante todo el intervalo, variando entre 143 y 178 municipios. La proporción de contactos examinados de casos nuevos en condición “precaria” aumentó en un 28,8% entre los municipios durante el período.

**Conclusión:**

La lepra permanece hiperendémica en el estado, con altas tasas de detección en menores de 15 años, una elevada proporción de casos con grado 2 de discapacidad física al diagnóstico y debilidades en las acciones de vigilancia y control. La elevada estimación de casos ocultos refuerza la necesidad de estrategias orientadas a ampliar la detección temprana y fortalecer la Atención Primaria.

**Ethical aspects**

This research respected ethical principles, having obtained the following approval data:

Research ethics committee Pesquisa da Universidade Federal do Piauí

Opinion number 6,793,557

Approval date 29/4/2024

Certificate of submission for ethical appraisal 78940424.9.0000.5214

Informed consent form Exempt

## Introduction

Hansen’s disease is a condition of significant historical and social impact, marked by stigma and prejudice. It is part of the Neglected Tropical Diseases group, as it is prevalent in tropical climates. Like other communicable diseases, several determining factors, especially socioeconomic ones, influence Hansen’s disease; therefore, it is considered a Socially Determined Disease. Its incidence is notably higher in developing countries, where social disparities and poor housing and hygiene conditions increase the risk of transmission and illness and pose a significant obstacle to diagnosis and treatment, given the difficulty of accessing health services [1,2].

In 2022, the global detection rate of Hansen’s disease was 21.8 cases per million inhabitants (174,087 new cases), concentrated mainly in India, Brazil, and Indonesia. In the same year, Brazil reported a rate of 9.67 cases per 100 thousand inhabitants (19,635 cases), remaining among the countries with the highest burden of disease. In Piauí, between 2013 and 2022, 8,838 cases were reported, with the highest detection rate recorded in 2017 (33.27/100,000 inhabitants) and the lowest in 2020 (16.27/100,000 inhabitants), placing it among the states with the highest rates in the Northeast [3,4].

It is important to emphasize that epidemiological studies from 2020 onward were significantly affected by the COVID-19 pandemic, particularly by a reduction in the identification of new cases. However, this decrease does not reflect a real decline in the number of new diagnoses, but rather limitations imposed on case-finding and detection strategies during the pandemic [3,4].

Given the epidemiological relevance of Hansen’s disease in the country, the continuous implementation of public health strategies is crucial for disease control. Key factors in this management include the provision of multidrug therapy by the Brazilian Unified Health System (*Sistema Único de Saúde*, SUS), the evaluation of contacts, and investments in access to the health care network. However, the country has not yet achieved the target (less than one case per 10 thousand inhabitants) for the elimination of Hansen’s disease as a public health problem, established by the World Health Organization [5,6].

Intending to expand the scope of existing control measures, the World Health Organization launched the Global Leprosy Strategy 2021–2030, seeking to strengthen initiatives to eliminate the disease and the disabilities it causes through early detection, immediate treatment, and the fight against stigma and discrimination [7]. In Brazil, the Ministry of Health created the National Strategy for Tackling Hansen’s Disease 2019–2022, aiming to reduce the impact of the condition by planning and implementing actions across the country’s three administrative levels. More recently, the National Strategy for Tackling Hansen’s Disease 2024-2030 was developed to resume the targets proposed by the previous plan, which were negatively affected by the pandemic [8,9]. 

To monitor and evaluate the actions implemented, surveillance uses epidemiological and operational indicators. The former monitors progress toward eliminating the disease as a public health problem by tracking new cases. Among the most relevant are the detection rate of new cases in individuals under 15 years of age and the proportion of new cases with grade 2 physical disability at diagnosis. The former measures the strength of endemic transmission, and the latter allows assessment of the effectiveness of timely case detection actions [10].

Operational indicators express the quality of health services, such as the proportion of contacts examined among those registered in the cohort years of new cases, used to assess contact surveillance. These indicators are relevant because they are associated with active disease transmission, late detection, factors related to the occurrence of disabilities, and the effectiveness of active case finding. The joint analysis of these indicators and the estimate of hidden prevalence guides strategies for addressing Hansen’s disease [4,11].

The concept of hidden prevalence of Hansen’s disease was introduced by Suárez and Lombardi in 1997 and involves estimating the number of undiagnosed cases in a population. For this procedure, case detection data from the five years preceding the year of interest are used, allowing inference of the existence of hidden cases [12].

The persistence of Hansen’s disease at endemic levels underscores the need for studies assessing the status of its indicators in the state, to encourage the implementation of more effective strategies to control its spread. Therefore, the objective of this study is to analyze epidemiological and operational indicators and the hidden endemicity of Hansen’s disease in Piauí from 2018 to 2022.

In line with the proposed objective, the research question was: how did the epidemiological and operational indicators of Hansen’s disease and hidden endemicity behave in Piauí from 2018 to 2022?

## Methods

### Study design

A descriptive study with spatial analysis was conducted using data compiled by the municipality in the state of Piauí.

### Setting and participants

Aggregated data on indicators of new cases of Hansen’s disease in Piauí, reported between 2018 and 2022, were used. Data were collected in May 2024, and all new cases of Hansen’s disease diagnosed in the state between 2018 and 2022 were included. 

For each variable analyzed, records with incomplete or inconsistent data were excluded only from the specific analysis of that variable, while available information was preserved for the remaining variables. This approach maximized the use of available data while minimizing information loss, without compromising the validity of the analyses.

The state of Piauí has 224 municipalities, distributed across 11 development territories (*territórios de desenvolvimento*, TD), which were institutionalized in 2007 and updated in 2017 through state legislation. Each territory has a Regional Health Coordination Office, equipped with its own administrative, technical, and political structure, and linked to the Secretary of State for Health of Piauí [13]. This territorial division was adopted in this study to represent the results cartographically, facilitating the visualization of indicator performance across regions.

### Variables

The variables analyzed comprised two epidemiological indicators (detection rate of new cases in individuals under 15 years of age per 100 thousand inhabitants and proportion of new cases with grade 2 physical disability at the time of diagnosis (%)) and one operational indicator (proportion of contacts examined among those registered in the cohort years of new cases (%)) [3,4].

### Data sources

The data source was the Hansen’s disease information database of the State Health Department of Piauí. This database contains absolute data on new cases, contacts, and information on physical disability for the period 2018–2022, provided directly by the Department for research purposes.

### Statistical methods

A descriptive analysis of the data obtained was performed. To measure the indicators, absolute values were converted to rates and percentages using the formulas established by the Ministry of Health [3,4]. Therefore, the detection rate of new cases in individuals under 15 years of age was calculated by dividing the number of cases in this age group by the corresponding population and multiplying by 100,000. The proportion of new cases with grade 2 physical disability corresponded to the number of cases with grade 2 disability divided by the total number of cases evaluated for degree of disability, multiplied by 100. The proportion of contacts examined was calculated by dividing the number of contacts examined by the total number registered, then multiplying by 100. Absolute data were also used to estimate hidden prevalence.

The absolute frequencies of municipalities in the state of Piauí for each indicator category of interest were recorded across the 2018–2022 time series (Table 1). To evaluate the data, the Ministry of Health established percentage parameters for each indicator, along with the corresponding classification. For the detection rate of new cases of Hansen’s disease in individuals under 15 years of age, the parameters are defined as follows: low, <0.50 per 100 thousand inhabitants; medium, 0.50–2.49 per 100 thousand inhabitants; high, 2.50–4.99 per 100 thousand inhabitants; very high, 5.00–9.99 per 100 thousand inhabitants; and hyperendemic, ≥10.00 per 100 thousand inhabitants [3].

**Table 1:** Distribution of new Hansen’s disease cases by year of notification and indicators. Piauí, 2018–2022 (n=3,789)

Indicator	2018	2019	2020	2021	2022	Total
New cases	1,027	865	535	679	692	3.789
Assessed	919	778	473	592	627	3,389
Disability grades 1 and 2	259	231	131	231	277	1,129
Percentage of cases with disability (c/b)^a^	28.18	29.69	27.69	39.02	44.17	-
Estimated number of undiagnosed cases (d/a)^b^	289	257	148	265	306	1,265

^a^The percentage of cases with disability among those assessed; ^b^ estimated number of undetected cases per year. The estimated hidden prevalence corresponds to the sum of the annual estimated cases over the five years.

The parameters for the proportion of new cases of Hansen’s disease with grade 2 physical disability at the time of diagnosis, reflecting the presence of disabilities and deformities, are as follows: low (≤5.0%), medium (5.0–9.9%), and high (≥10.0%). The indicator for the proportion of contacts examined for new diagnoses is measured as follows: good (≥90.0%); fair (75.0–89.9%); and poor (<75.0%) [3]. For each parameter, the percentage change between the first and the last year was also calculated using the following equation:


Percentage change=[(final value−
initial value)÷initial value]×100

Due to fluctuations in variable values throughout the series, this percentage does not provide an assessment applicable to isolated years, but rather to the five years as a whole.

Based on the proposal by Suárez and Lombardi [12], the indicators for each year of the time series analyzed in this study were recorded (Table 2). The following indicators were considered: number of new cases; number of cases evaluated; number of individuals with disability (Grades 1 and 2); percentage of individuals with disability; and estimated number of undetected cases. In addition, hidden prevalence was calculated and estimated using the following relationship:

**Table 2:** Number of municipalities for each indicator parameter by year. Piauí, 2018–2022 (n=224)

Parameter	2018	2019	2020	2021	2022	Variation (%)
**Indicator: Detection rate of new cases in individuals under 15 years of age**						
Low (<0.50 per 100 thousand inhabitants)	195	205	205	210	209	+7.17
Medium (0.50–2.49 per 100 thousand inhabitants)	0	0	0	1	0	0
High (2.50–4.99 per 100 thousand inhabitants)	1	1	2	0	1	0
Very high (5.00-9.99 per 100 thousand inhabitants)	1	1	1	2	3	+200
Hyperendemic (≥10.00 per 100 thousand inhabitants)	27	17	16	11	11	-59.25
**Indicator: Proportion of new cases with grade 2 physical disability at the time of diagnosis**						
Low (≤5.0%)	143	164	178	159	155	+8.39
Medium (5.0–9.9%)	0	1	1	0	2	0
High (≥10.0%)	81	59	45	65	67	-17.28
**Indicator: Proportion of contacts examined among those registered in the cohorts of new cases**						
Good (≥90.0%)	68	55	50	49	39	-42.64
Fair (75.0-89.9%)	22	14	7	13	12	-45.45
Poor (<75.0%)	134	155	167	162	174	+29.85


Estimated hidden prevalence=(number of cases with physical disabilities at the time of diagnosis among those evaluated÷number of cases evaluated at the time of diagnosis)×total number of new cases diagnosed in the year.

For each of the five years in the sample, the percentage of newly diagnosed cases presenting any disability (among those evaluated for disability) was calculated [12]. This percentage was applied to the total number of new cases detected in the same year, yielding an estimate of the number of undetected cases. Therefore, the sum of the results for the five years corresponds to the estimated hidden prevalence. 

After the analyses, cartographic representations of the results were created using the open-source geoprocessing software Quantum GIS version 3.36.3 Maidenhead, to provide a spatial perspective of the indicators analyzed throughout the time series. The study did not include inferential statistical analyses or other more robust analytical approaches, such as correlation tests or temporal trend analyses.

## Results

During the study period (2018–2022), 3,789 new cases of Hansen’s disease were reported (Table 1). The highest number of diagnoses occurred in 2018 (n=1,027), with a detection rate of 32.93 cases per 100 thousand inhabitants. Subsequently, a steady reduction was observed until 2020 (n=535). In the following years, the number of notifications increased to 679 in 2021 and 692 in 2022.

Of the total number of diagnosed cases, 3,389 were evaluated for physical disability. The evaluation percentage ranged from 87.1% (2021) to 90.0% (2022). Overall, 1,129 individuals had some degree of disability, with the highest number of cases in 2018 (n=259), followed by 2019 and 2021 (n=231 in both years).

The estimated hidden prevalence calculation suggests a total of 1,265 undiagnosed cases over the five years studied. This number, added to the total registered notifications, suggests a total of 5,054 new cases of Hansen’s disease over the time series.

A 59.2% reduction in the number of municipalities classified as hyperendemic for the detection rate among individuals under 15 years of age was observed, decreasing from 27 in 2018 to 11 in 2021 and 2022 (Table 2). Additionally, fluctuations in parameter distribution were observed over the years, yet all 11 health regions had municipalities with high endemicity in at least one year of the interval (Figure 1).

**Figure 1:**
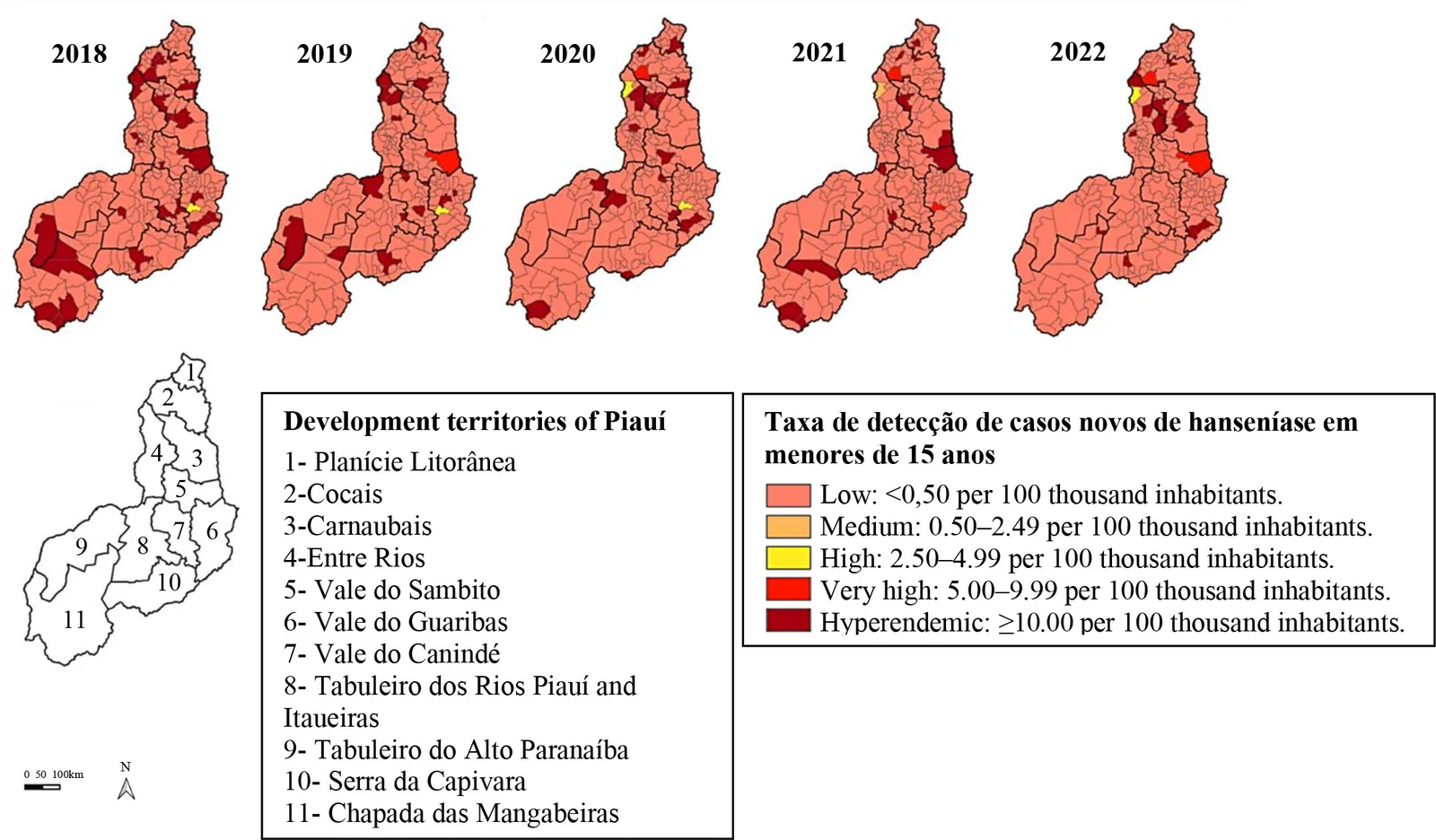
Spatial distribution of the proportion of new Hansen’s disease cases identified in individuals under 15 years of age by development region. Piauí, 2018–2022 (n=224)

Regarding the spatial distribution of the parameters of the indicator proportion of new cases of Hansen’s disease with Grade 2 physical disability at the time of diagnosis, it was observed that the majority of municipalities (between 143 and 178) were classified as “low” throughout the entire period analyzed. All 11 regions had municipalities with this parameter (Figure 2), and the highest number occurred in 2020 (n=178).

**Figure 2:**
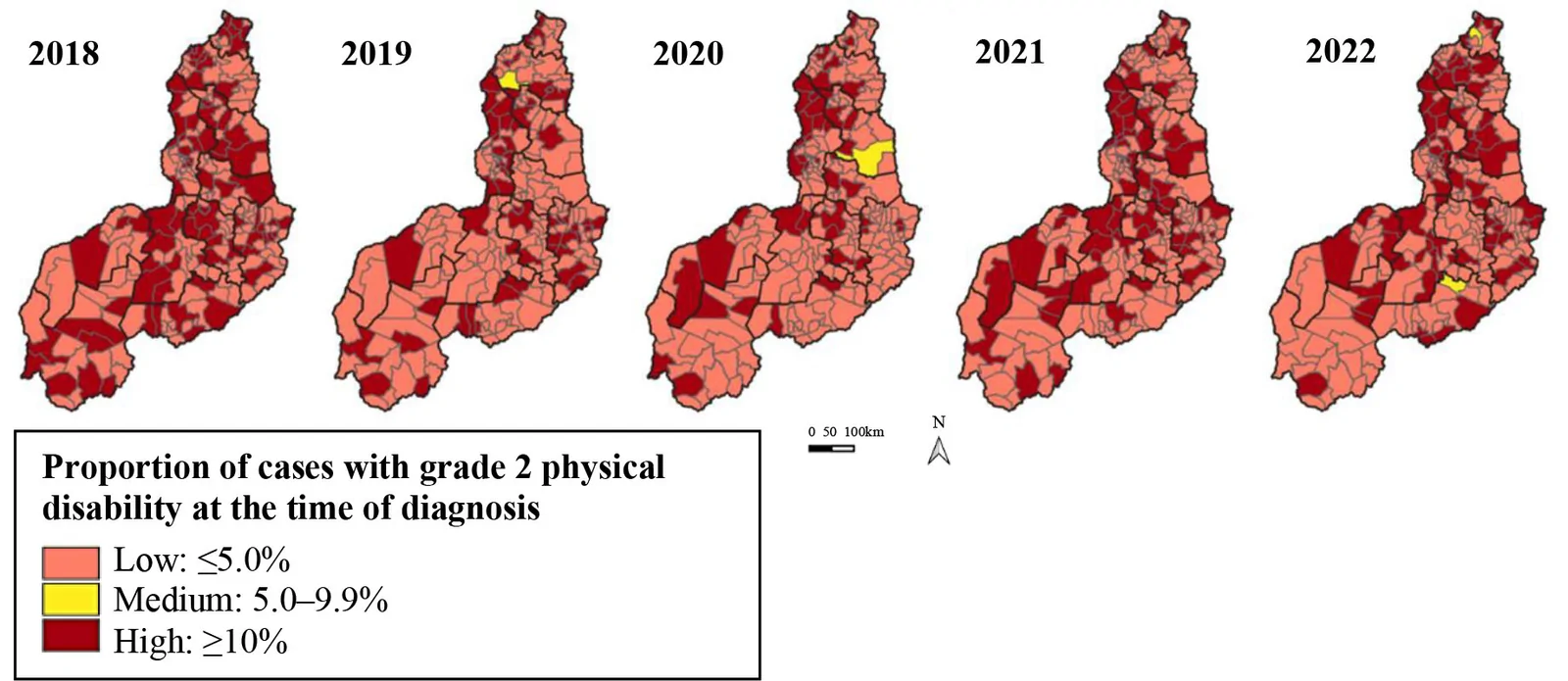
Spatial distribution of the indicator of the proportion of cases with grade 2 physical disability at the time of diagnosis by development region. Piauí, 2018–2022 (n=224)

The number of municipalities with high incidence decreased over the years, from 81 in 2018 to 67 in 2022. The lowest number was recorded in 2020, with 45 municipalities. However, an increase was observed in the two subsequent years (65 in 2021 and 67 in 2022). The opposite pattern was observed compared to municipalities classified in the “low” parameter, in which there was a reduction from 178 to 159 and then to 145. The “medium” parameter fluctuated from zero to two municipalities during the interval.

Regarding the spatial distribution of the indicator proportion of contacts examined among new cases of Hansen’s disease diagnosed in the years analyzed, a reduction in the number of municipalities classified as “good” was observed, from 68 in 2018 to 39 in 2022. Conversely, the “poor” parameter increased by 29.8%, rising from 134 municipalities at the beginning of the interval (2018) to 174 at the end (2022). The number of municipalities with “fair” classification decreased in 2018 (n=22), 2019 (n=14), and 2022 (n=7), and increased in 2022 (n=13). It reached 12 municipalities in the final year (Figure 3).

**Figure 3:**
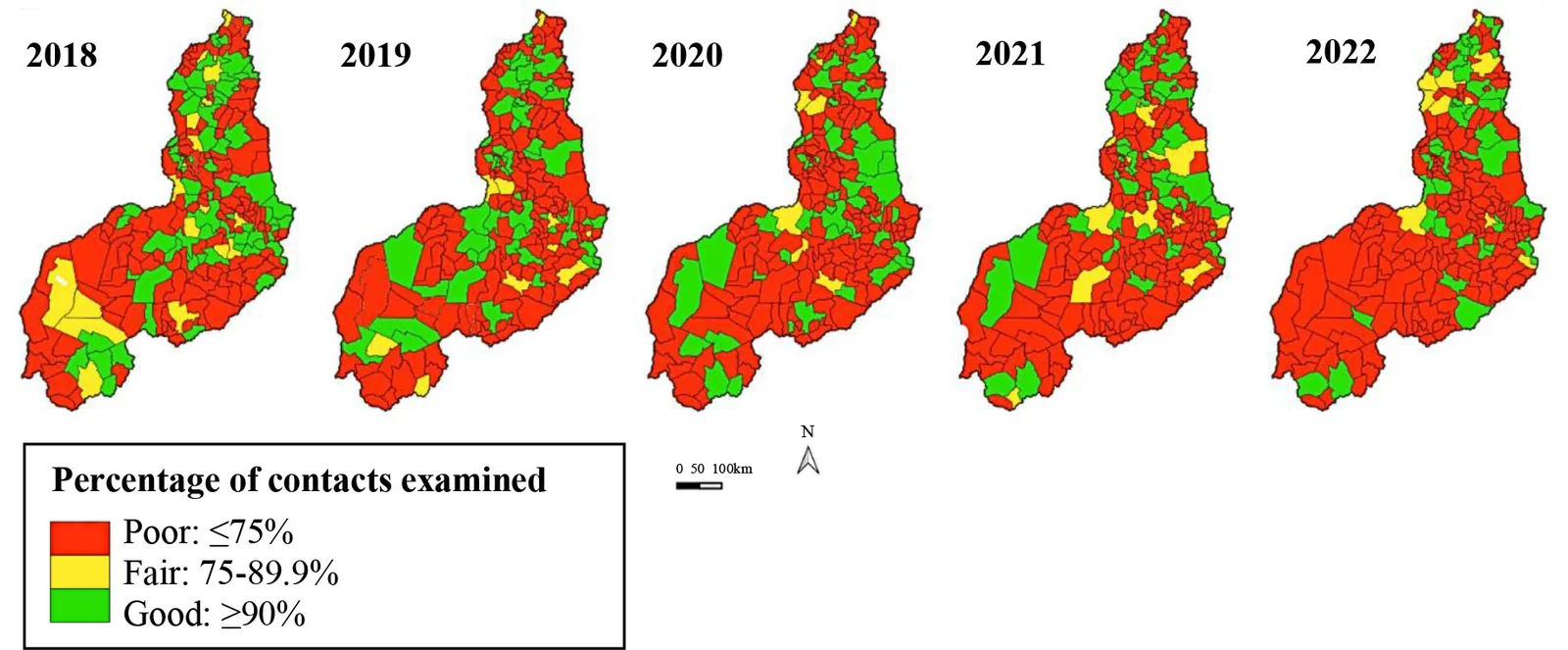
Spatial distribution of the indicator of the proportion of contacts of new diagnoses assessed by development region. Piauí, 2018–2022 (n=224)

## Discussion

The analysis of the data over the five years revealed variations in the indicators monitored, with fluctuations in both the number of new cases and the other observed values. A reduction in diagnoses was identified in 2020, the first year of the COVID-19 pandemic, followed by increases in subsequent years. The calculation of hidden prevalence suggests that a larger number of cases may exist, possibly undetected during the period. The evaluation of the indicators shows an improvement in the hyperendemic status of the disease among individuals under 15 years of age, but a setback in the proportion of contacts examined. In turn, the proportion of new cases with disabilities presents a more uncertain scenario, due to the effects of the pandemic, which make it difficult to infer with certainty a trend of improvement, worsening, or maintenance of the situation. These findings reflect possible weaknesses in Hansen’s disease surveillance and case-finding actions.

Between 2007 and 2021, the highest prevalence of cases of the disease in the state was recorded in Teresina, the capital located in the Entre Rios territory. High incidences were also observed in the regions of Vale do Rio Guaribas, Vale dos Rios Piauí, and Itaueiras [14,15]. 

In Brazil, also between 2018 and 2022, 112,456 new cases of the disease were reported, with the highest number of diagnoses in Mato Grosso, Maranhão, Tocantins, Goiás, and Piauí [4]. The data from Piauí revealed in our study suggest an alarming status; however, it should also be considered that there are failures in the diagnostic process, which may result in underreporting [16,17]. Possible explanations include lack of knowledge and stigma among the population, shortcomings in professional training, and inadequate coverage of contact evaluation, suggesting that the actual number of cases is much higher than that identified [18].

In addition, the study period was affected by the COVID-19 pandemic. During this period, a reduction in the detection rate per 100 thousand inhabitants was observed, with the lowest value recorded in the year the pandemic began (16.2 in 2020). This reduction in the rate in 2020 was also observed in São Paulo and throughout Latin America [19,20]. In the context of the pandemic, the overload of the health system and the redirection of resources toward coronavirus control and response, with a consequent reduction in diagnostic and reporting activities for Hansen’s disease, may have contributed to this decline [11,21].

Between 2001 and 2015, more than 240 thousand new cases of Hansen’s disease were reported in the Northeast region, of which 8.7% occurred in children aged 0 to 14 years. During this period, only the states of Piauí, Paraíba, and Bahia showed a decline in detection [22]. In this analysis, the number of municipalities classified as hyperendemic for this indicator continued to decrease. At the beginning of the interval (2018), the regions showed an almost uniform distribution of this status. Except for Planície Litorânea, all regions had between one and three municipalities in this category. By the end of the period (2022), however, no municipalities with this classification were observed in the regions of Planície Litorânea, Chapada das Mangabeiras, Tabuleiro do Alto Parnaíba, Tabuleiro dos Rios Piauí e Itaueiras, Vale do Canindé, and Vale do Sambito.

Between 2007 and 2021, in Piauí, the detection rate among individuals under 15 years of age decreased, although the average remained hyperendemic in this age group [14]. In our analysis, we observed a 59% reduction in the number of municipalities classified as hyperendemic for the same rate (from 27 to 11), alongside a 7% increase in those described as “low” (from 195 to 209). Although detection has declined over time, some municipalities still exhibit hyperendemic levels.

The detection of cases in this age group (<15 years) is an important indicator of endemicity, as it reveals foci of active transmission where children are exposed to affected individuals or to inadequately treated cases [23]. Hansen’s disease can generate significant impacts on children and adolescents in the physical, emotional, and social domains, since the damage caused by the disease may interfere with body image and self-esteem through physical disabilities. This may negatively influence identity development and social relationships, compromise school performance, and even lead to school dropout due to discrimination [24].

The Degree of Physical Disability is defined by the Simplified Neurological Assessment (*Avaliação Neurológica Simplificada*, SNA), which assesses neural impairment. The results may be: grade 0 for absence; grade 1 for decreased or loss of sensitivity; and grade 2 for the presence of disabilities and deformities [10]. In the time series analyzed, only in 2022 did the state evaluate at least 90.0% of new disability cases. This suggests a regression scenario, since between 2001 and 2015, Piauí and Sergipe were the only states in the Northeast to achieve an average above this same percentage [22].

In view of this, a reduction in the number of individuals with grade 2 physical disability at diagnosis can be observed until 2020. The Chapada das Mangabeiras region stands out in this pattern, reducing the number of municipalities with high detection rates by half (from 8 to 4). The opposite result is observed at the national level, where a constant increase has been observed since 2014 [4]. Despite this local reduction, the findings suggest a possible service failure in disability assessment, which is not performed in all patients at the time of diagnosis.

It is known that grade 2 physical disability at diagnosis indicates late detection. Thus, this indicator also serves as an indirect indicator of hidden endemicity and allows estimation of a more realistic prevalence [11,21]. Based on the calculation of hidden prevalence during the study period, it is possible to estimate that the number of underreported cases increased to 1,265 (Table 1). In 2022, the hidden number accounted for almost half the reported cases (306 unreported cases compared with 692 recorded). Considering the long incubation period of Hansen’s disease, this estimate does not necessarily indicate new infections in that specific year, but rather cases that progressed until the development of disabilities.

In this sense, contact examination functions as a strategy for active case detection. The indicator that analyzes the proportions of these evaluations reflects the capacity of health services to conduct surveillance. Considering the epidemiological importance of contacts in Hansen’s disease and the need for early diagnosis, low numbers may indicate limited access to services and health actions, hindering disease control and contributing to hidden endemicity and late diagnosis [25].

The findings of this study suggest that contact evaluation during the analyzed period showed a consistent worsening, evidenced by a 29.8% increase in the number of municipalities classified as “poor” for this indicator, with absolute growth in all years of the sample. This finding differs from the 2024 estimate for the state of Amazonas. At that time, a positive progression in the proportion of contacts examined in the state was observed, varying from 73% to 88% between 2012 and 2021 [26]. In 2022, the precarious situation of this indicator was observed almost uniformly across municipalities in the southern region of the state. In the same year, Piauí ranked third among the states with the most municipalities meeting this parameter, with 57 cities, behind only Bahia (106) and Minas Gerais (78) [4].

This weakness in contact evaluation may be attributed to the stigma associated with Hansen’s disease, which generates fear both of receiving the diagnosis and of communicating it to family members. In addition, other factors, such as limited health education, difficulties in accessing services, and shortcomings in the performance of health teams, contribute to the late diagnosis of the disease [25].

Within the scope of the National Strategy for Tackling Hansen’s Disease 2019–2022, the Ministry of Health established key targets, such as reducing the detection rate among individuals under 15 years of age and the proportion of new cases with grade 2 disability, as well as combating discriminatory practices against affected individuals and their families, with the main objective of reducing the burden of the disease in the country [8]. Subsequently, the Strategy for the period 2024–2030 reinforced these axes. It defined new targets, such as interrupting transmission in a large part of the national territory, eliminating Hansen’s disease in 75.0% of municipalities, reducing by 20.0% the absolute number of new cases with grade 2 disability at the time of diagnosis, and providing comprehensive response to 100% of complaints regarding discriminatory practices recorded in the SUS Ombudsman Office [9].

In this study, it was observed that detection rates among individuals under 15 years of age remained high and that the proportion of cases with disability at diagnosis remained substantial, indicating, up to the temporal limit of the analysis, a mismatch with the strategic targets. These findings reinforce the need to intensify the actions planned for the period 2024–2030, particularly in strengthening timely detection, active contact tracing, and improving notification processes.

In this context, primary health care plays a central role in controlling Hansen’s disease. Primary health care units carry out surveillance and prevention activities in a decentralized manner, involving a multiprofessional team that must be trained to recognize signs and symptoms and monitor active cases. Active case finding and contact surveillance enable early diagnosis, preventing disabilities and complications. In addition, health education is integrated into primary health care strategies, facilitating population recognition of disease and contributing to treatment adherence and stigma reduction [27].

Primary health care professionals, especially nurses, play an indispensable role in identifying cases of Hansen’s disease, providing follow-up, conducting contact evaluations, and encouraging self-care. The control and management of the disease should be regarded as a permanent task, taking into account the specificities of each population. Investment in epidemiological surveillance, prevention, active case finding, and monitoring of confirmed cases should be considered, along with support from referral services responsible for providing matrix support [3,28].

It is important to note that this study has limitations related to the quality and reliability of the information, which may be subject to inconsistencies in the notification and recording processes. As secondary data were used, there is the possibility of underreporting in information systems, as well as limitations in interpreting the so-called hidden prevalence. Furthermore, the study had an exclusively descriptive design and did not include more robust statistical analyses.

 The analyses were conducted at the state level, which limits the interpretation of local variations and may lead to ecological fallacy. Despite these limitations, the findings provide a comprehensive view of the distribution and evolution of epidemiological and operational indicators of Hansen’s disease in Piauí, supporting planning and disease control actions and encouraging further studies focused on time series and regional analyses. 

The values of the analyzed indicators show that Hansen’s disease remains classified as hyperendemic in the state, marked by high detection rates among individuals under 15 years of age, a high proportion of cases with grade 2 disability, and weaknesses in surveillance and control actions. The large, estimated number of hidden cases underscores the need for strategies that expand early detection capacity, strengthen primary health care, and improve notification processes to support the promotion, planning, and implementation of coordinated actions across the three levels of government. This is crucial for strengthening control of the endemic disease in the state, aiming to reduce transmission and disease-related complications.

## Data Availability

The data used in this study are available at https://osf.io/pq5w3/.
