# Supplementary material for: Indicators, spatial distribution, and hidden endemicity of Hansen’s disease: descriptive study, Piauí, 2018–2022
Source: Epidemiol Serv Saude. 2026 Jul 20;35:e20250504. doi: 10.1590/S2237-96222026v35e20250504.en (PMC13686427; doi:10.1590/S2237-96222026v35e20250504.en)
Supplement: Translation

# Translation: Indicadores, distribuição espacial e endemia oculta da hanseníase: estudo descritivo, Piauí, 2018–2022

**DOI:** 10.1590/S2237-96222026v35e20250504.pt

**Authors:** Khaab Gibran Leal Vasconcelos, Lara Beatriz de Sousa Araújo, Raimundo Leoberto Torres de Sousa, Olívia Dias de Araújo

**Affiliations:** 1 Universidade Federal do Piauí Teresina Teresina PI Brasil Universidade Federal do Piauí, Teresina, PI, Brasil; 2 Universidade Federal do Piauí Programa de Pós-Graduação em Saúde e Comunidade Teresina PI Brasil Universidade Federal do Piauí, Programa de Pós-Graduação em Saúde e Comunidade, Teresina, PI, Brasil; 3 Centro de Inteligência em Agravos Tropicais Emergentes e Negligenciados Teresina Teresina PI Brasil Centro de Inteligência em Agravos Tropicais Emergentes e Negligenciados, Teresina, PI, Brasil

**Keywords:** Hanseníase, Indicadores Básicos de Saúde, Indicadores de Serviços, Saúde Pública, Estudos Epidemiológicos

## Abstract

**Objetivo:**

Analisar indicadores epidemiológicos e operacionais e a endemia oculta de hanseníase no Piauí, no período de 2018 a 2022.

**Métodos:**

Estudo descritivo, com análise espacial, utilizando dados fornecidos pela Secretaria de Estado da Saúde do Piauí. As variáveis analisadas foram: taxa de detecção de casos novos em menores de 15 anos; proporção de casos novos com grau 2 de incapacidade física no diagnóstico e percentual de avaliação de contatos de novos diagnósticos. Os indicadores foram apresentados em frequência absoluta por município, com cálculo da variação percentual. Foram elaborados mapas temáticos e estimada a prevalência oculta da hanseníase no estado.

**Resultados:**

Identificaram-se 3.789 novos casos. Pela prevalência oculta, estima-se 1.265 casos não diagnosticados no período. Em 2018, 27 municípios eram considerados hiperendêmicos quanto à detecção em menores de 15 anos, reduzindo para 11 ao final da série. A distribuição de casos novos com grau de incapacidade 2 no diagnóstico apresentou-se majoritariamente no parâmetro “baixo” durante o intervalo, variando de 143 a 178 municípios. A proporção de contatos examinados de casos novos em status “precário” aumentou em 28,8% o número de municípios no período.

**Conclusão:**

A hanseníase permanece hiperendêmica no estado, com altas taxas de detecção em menores de 15 anos, elevada proporção de casos com grau 2 de incapacidade física no diagnóstico e fragilidades nas ações de vigilância e controle. A elevada estimativa de casos ocultos reforça a necessidade de estratégias voltadas à ampliação da detecção precoce e ao fortalecimento da Atenção Primária.

**Aspectos éticos**

Esta pesquisa respeitou os princípios éticos, obtendo os seguintes dados de aprovação:

Comitê de ética em pesquisa Universidade Federal do Piauí

Número do parecer 6.793.557

Data de aprovação 29/4/2024

Certificado de apresentação de apreciação ética 78940424.9.0000.5214

Registro do consentimento livre e esclarecido Dispensado

## Introdução

A hanseníase é um agravo de relevante impacto histórico e social, marcado por estigma e preconceito. Ela integra o grupo das Doenças Tropicais Negligenciadas, uma vez que é considerada uma doença prevalente em países de clima tropical. Assim como outras condições transmissíveis, diversos fatores determinantes, sobretudo socioeconômicos, influenciam a hanseníase; por isso é considerada uma Doença Determinada Socialmente. Sua incidência é notoriamente mais alta em países em desenvolvimento, onde as disparidades sociais e condições de moradia e higiene aumentam o risco de transmissão e adoecimento e se tornam um obstáculo significativo no diagnóstico e tratamento, dada a dificuldade de acesso aos serviços de saúde [1,2].

Em 2022, a taxa global de detecção de hanseníase foi de 21,8 casos por milhão de habitantes (174.087 casos novos), concentrados majoritariamente na Índia, no Brasil e na Indonésia. No mesmo ano, o Brasil apresentou uma taxa de 9,67 casos por 100 mil habitantes (19.635 casos), permanecendo entre os países de maior carga da doença. No Piauí, entre 2013 e 2022, foram notificados 8.838 casos, com a maior taxa de detecção registrada em 2017 (33,27/100 mil habitantes) e a menor em 2020 (16,27/100 mil habitantes), situando-se entre os estados com maiores taxas do Nordeste [3,4].

É importante ressaltar que os levantamentos epidemiológicos a partir de 2020 sofreram impacto significativo com a pandemia de covid-19, resultando, especialmente, na redução da identificação de novos casos. No entanto, tal diminuição não significa queda real no número de novos diagnósticos, mas sim limitações impostas às estratégias de busca e de detecção diante do cenário pandêmico [3,4].

Dada a relevância epidemiológica da hanseníase no país, implementar continuamente estratégias de saúde pública é crucial para o controle da doença. Consideram-se fatores-chave nesse manejo o fato de o Sistema Único de Saúde disponibilizar a poliquimioterapia e os contatos e os investimentos no acesso à rede de saúde serem avaliados. No entanto, o país ainda não atingiu a meta (menos de um caso por 10 mil habitantes) de eliminação da hanseníase como problema de saúde pública, estabelecida pela Organização Mundial da Saúde [5,6].

Com o objetivo de ampliar a abrangência das medidas de controle vigentes, a Organização Mundial de Saúde lançou *Estratégia Global de Hanseníase 2021*-2030 buscando impulsionar as iniciativas voltadas para a eliminação da doença e de incapacidades ocasionadas, por meio da detecção precoce, do tratamento imediato e do combate ao estigma e à discriminação [7]. No Brasil, o Ministério da Saúde formulou a *Estratégia Nacional para Enfrentamento da Hanseníase 2019*-2022, visando diminuir o impacto do agravo no país a partir do planejamento e da execução de ações nas três esferas administrativas. Mais recentemente, foi formulada a *Estratégia organizada para os anos 2024*-2030, com a finalidade de retomar as metas propostas pelo plano anterior, que foram afetadas negativamente pela pandemia [8,9]. 

Para acompanhar e avaliar as ações realizadas, a vigilância utiliza indicadores epidemiológicos e operacionais. Os primeiros monitoram o progresso na eliminação da doença como problema de saúde pública, a partir do número de novos casos. Entre os mais relevantes, estão a taxa de detecção de casos novos em menores de 15 anos e a proporção de casos novos com grau 2 de incapacidade física no diagnóstico. A primeira mensura a força da transmissão endêmica e a segunda permite avaliar a efetividade das ações de detecção oportuna [10].

Os indicadores operacionais expressam a qualidade dos serviços de saúde, a exemplo da proporção de contatos examinados de casos novos nos anos das coortes, utilizada para avaliar a vigilância de contatos. Esses indicadores são relevantes por estarem associados à transmissão ativa da doença, bem como à detecção tardia, fatores relacionados à ocorrência de incapacidades e à efetividade da busca ativa. A análise conjunta desses indicadores e da estimativa de prevalência oculta orienta as estratégias de enfrentamento da hanseníase [4,11].

O conceito de prevalência oculta da hanseníase foi introduzido por Suárez e Lombardi em 1997 e consiste em um cálculo estimativo do número de casos ainda não diagnosticados em uma população. Para tal operação, utilizam-se dados de detecção de casos nos cinco anos antecedentes ao ano de avaliação desejado, permitindo inferir a existência de casos ocultos [12].

A manutenção da hanseníase em nível endêmico evidencia a necessidade de estudos que abordem a situação dos indicadores do agravo no estado, com a finalidade de estimular a implementação de estratégias mais eficazes para controlar a disseminação da hanseníase. Portanto, o objetivo deste estudo é analisar indicadores epidemiológicos e operacionais e a endemia oculta de hanseníase no Piauí, no período de 2018 a 2022.

Em consonância com o objetivo proposto, a questão de pesquisa que se buscou responder foi: de que forma os indicadores epidemiológicos e operacionais da hanseníase e a endemia oculta se comportaram no Piauí no período de 2018 a 2022?

## Métodos

### Delineamento do estudo

Estudo descritivo, com análise espacial, realizado com dados compilados por município do estado do Piauí.

### Contexto e participantes

Foram utilizados dados agregados de indicadores relacionados a casos novos de hanseníase no Piauí notificados entre 2018 e 2022. Os dados foram coletados no mês de maio de 2024 e foi considerado como critério de inclusão todos os casos novos de hanseníase diagnosticados no estado entre 2018 e 2022. 

Para cada variável analisada, registros com dados incompletos ou inconsistentes foram excluídos apenas da análise específica daquela variável, preservando-se as informações disponíveis para as demais variáveis. Essa abordagem permitiu aproveitar ao máximo os dados disponíveis, minimizando perdas de informação sem comprometer a validade das análises.

O estado do Piauí tem 224 municípios, distribuídos em 11 territórios de desenvolvimento, cuja institucionalização ocorreu em 2007 e foi atualizada em 2017 por meio de legislação estadual. Cada território dispõe de uma Coordenação Regional de Saúde, dotada de estrutura administrativa, técnica e política próprias, e vinculada à Secretaria de Estado da Saúde do Piauí [13]. Essa divisão territorial foi adotada neste estudo para representar de forma cartográfica os resultados, com o objetivo de facilitar a visualização do desempenho dos indicadores em cada região.

### Variáveis

As variáveis trabalhadas foram dois indicadores epidemiológicos (taxa de detecção de casos novos em menores de 15 anos por 100 mil habitantes e proporção de casos novos com grau 2 de incapacidade física no momento do diagnóstico (%)) e um operacional (proporção de contatos examinados dentre os registrados nos anos das coortes de novos casos (%)) [3,4].

### Fonte de Dados

A fonte de dados foi o banco de informações sobre hanseníase da Secretaria de Estado da Saúde do Piauí. Esse banco contém dados absolutos de casos novos, contatos e informações sobre incapacidade física, referentes ao período de 2018 a 2022, fornecidos diretamente pela Secretaria para fins de pesquisa.

### Métodos estatísticos

Foi realizada uma análise descritiva dos dados obtidos. Para a mensuração dos indicadores, os valores absolutos foram convertidos em taxas percentuais, de acordo com as fórmulas estabelecidas pelo Ministério da Saúde [3, 4]. Assim, a taxa de detecção de casos novos em menores de 15 anos foi calculada dividindo-se o número de casos nessa faixa etária pela população correspondente, multiplicado por 100 mil. A proporção de casos novos com grau 2 de incapacidade física correspondeu ao número de casos com grau 2 dividido pelo total de casos avaliados quanto ao grau de incapacidade, multiplicado por 100. Já a proporção de contatos examinados foi obtida pelo número de contatos examinados dividido pelo total registrado, multiplicado por 100. Os dados absolutos também foram utilizados para estimar a prevalência oculta.

Registrou-se a frequência absoluta do número de municípios do estado do Piauí em cada categoria dos indicadores de interesse ao longo da série temporal de 2018 a 2022 (Tabela 1). Para avaliar os dados, foram utilizados os percentuais dos parâmetros estabelecidos pelo Ministério da Saúde para cada indicador, bem como a classificação referida. Para a taxa de detecção de casos novos de hanseníase em menores de 15 anos, os parâmetros são considerados: baixo, <0,50 por 100 mil habitantes; médio, 0,50 a 2,49 por 100 mil habitantes; alto, 2,50 a 4,99 por 100 mil habitantes; muito alto, 5,00 a 9,99 por 100 mil habitantes; e hiperendêmico, ≥10,00 por 100 mil habitantes [3].

**Tabela 1:** Distribuição dos casos novos de hanseníase por ano de notificação e indicadores. Piauí, 2018-2022 (n=3.789)

Indicador	2018	2019	2020	2021	2022	Total
Casos novos	1.027	865	535	679	692	3.789
Avaliados	919	778	473	592	627	3.389
Incapacitados 1 e 2	259	231	131	231	277	1.129
Percentual de incapacitados (c/b)^a^	28,18	29,69	27,69	39,02	44,17	-
Estimativa de casos não diagnosticados (d/a)^b^	289	257	148	265	306	1.265

^a^Percentual de incapacitados entre os avaliados; ^b^Estimativa de casos não detectados para cada ano; A estimativa de prevalência oculta corresponde à soma do número estimado anual dos 5 anos do período.

Os parâmetros para a proporção de casos novos de hanseníase com grau 2 de incapacidade física no momento do diagnóstico, que correspondem à presença de incapacidades e deformidades, são descritos como: baixo, ≤5,0%; médio, 5,0 a 9,9%; e alto, ≥10,0%. O indicador de proporção de contatos examinados, referente a novos diagnósticos, é mensurado como: bom, ≥90,0%; regular, 75,0 a 89,9%; e precário, <75,0% [3]. Para cada parâmetro, também foi calculada a variação percentual entre o valor do primeiro e do último ano, utilizando a seguinte equação:


Variação percentual=[(Valor final−
Valor inicial)÷Valor inicial]×100

Devido às oscilações de valores das variáveis no decorrer da série, esse percentual não confere uma avaliação aplicável a anos isolados, mas sim ao quinquênio propriamente.

A partir da proposta de Suárez e Lombardi [12], registraram-se os indicadores definidos referentes a cada ano da série temporal analisada neste estudo (Tabela 2). Foram considerados os seguintes indicadores: número de casos novos; número de casos avaliados; número de indivíduos com incapacidade (graus 1 e 2); percentual de incapacitados; e estimativa de casos não detectados. Além disso, foi calculada e estimada a prevalência oculta, obtida a partir da seguinte relação:


Estimativa de prevalência oculta=(nº de casos com incapacidades físicas no momento do diagnóstico, entre aqueles avaliados÷nº de casos avaliados no momento do diagnóstico)×nº total de casos novos diagnosticados no ano.

**Tabela 2:** Número de municípios para cada parâmetro dos indicadores por ano. Piauí, 2018-2022 (n=224)

Parâmetro	2018	2019	2020	2021	2022	Variação (%)
**Indicador: Taxa de detecção de casos novos em menores de 15 anos**						
Baixo (<0,50 por 100 mil habitantes)	195	205	205	210	209	+7,17
Médio (0,50-2,49 por 100 mil habitantes)	0	0	0	1	0	0
Alto (2,50-4,99 por 100 mil habitantes)	1	1	2	0	1	0
Muito alto (5,00-9,99 por 100 mil habitantes)	1	1	1	2	3	+200
Hiperendêmico (≥10,00 por 100 mil habitantes)	27	17	16	11	11	-59,25
**Indicador: Proporção de casos novos com grau 2 de incapacidade física no momento do diagnóstico**						
Baixo (≤5,0%)	143	164	178	159	155	+8,39
Médio (5,0- 9,9%)	0	1	1	0	2	0
Alto (≥10,0%)	81	59	45	65	67	-17,28
**Indicador: Proporção de contatos examinados dentre os registrados nos anos das coortes de novos casos**						
Bom (≥90,0%)	68	55	50	49	39	-42,64
Regular (75,0-89,9%)	22	14	7	13	12	-45,45
Precário (<75,0%)	134	155	167	162	174	+29,85

Para cada um dos 5 anos da amostra, foi calculado o percentual de casos novos diagnosticados com alguma incapacidade (entre os que foram avaliados para incapacidade) [12]. Esse percentual foi aplicado ao total de casos novos detectados no mesmo ano, obtendo-se uma estimativa do número de casos não detectados referentes àquele ano. Dessa forma, a soma do resultado dos 5 anos corresponde ao estimado da prevalência oculta. 

Após as análises, foram criadas representações cartográficas com os resultados utilizando o software de geoprocessamento livre Quantum GIS versão 3.36.3 Maidenhead, com objetivo de oferecer uma perspectiva espacial dos indicadores analisados no decorrer da série temporal. O estudo não incluiu análises estatísticas inferenciais ou outras abordagens analíticas mais robustas, como testes de correlação ou de tendência temporal.

## Resultados

No período estudado (2018-2022) foram notificados 3.789 novos casos de hanseníase (Tabela 1). O maior número de diagnósticos foi em 2018 (n=1.027), com uma taxa de detecção de 32,93 casos/100 mil habitantes. Em seguida, observou-se redução constante até 2020 (n=535). Nos anos posteriores, o número de notificações aumentou para 679 em 2021 e 692 em 2022.

Do total de casos diagnosticados, 3.389 foram avaliados quanto à presença de incapacidade física. O percentual de avaliações flutuou entre 87,1% (2021) e 90,0% (2022). No total, 1.129 pessoas possuíam alguma incapacidade, com maior número de casos no ano de 2018 (n=259), seguido dos anos de 2019 e 2021 (n=231 em ambos).

O cálculo da estimativa de prevalência oculta sugere um total de 1.265 casos não diagnosticados no quinquênio estudado. Esse número, somado ao total de notificações registradas, sugere um quantitativo real de 5.054 casos novos de hanseníase na série temporal.

Foi observada uma redução do número de municípios em estado hiperendêmico para o indicador de taxa de detecção em menores de 15 anos de 59,2%, mudando de 27 em 2018 para 11 em 2021 e 2022 (Tabela 2). Ainda, percebeu-se uma flutuação do quantitativo dos parâmetros com o passar dos anos, mas todas as 11 regiões de saúde apresentaram cidades com alta endemia em pelo menos algum ano do intervalo (Figura 1).

**Figura 1:** Distribuição espacial da proporção do indicador de casos novos de hanseníase identificados em menores de 15 anos por região de desenvolvimento. Piauí, 2018-2022 (n=224)

Quanto à distribuição espacial dos parâmetros do indicador de proporção de casos novos de hanseníase com grau 2 de incapacidade física no momento do diagnóstico, observou-se que a maioria dos municípios (entre 143 e 178) apresentou classificação “baixo” durante todo o intervalo analisado. Todas as 11 regiões apresentaram cidades com esse parâmetro (Figura 2), e o maior quantitativo ocorreu no ano de 2020 (n=178).

**Figura 2:** Distribuição espacial do indicador de proporção do grau de incapacidade física 2 no momento do diagnóstico por região de desenvolvimento. Piauí, 2018-2022 (n=224)

O número de municípios com alta incidência reduziu com o passar dos anos, passando de 81 em 2018 para 67 em 2022. O menor quantitativo foi em 2020, com um total de 45 municípios. Entretanto, observou-se um aumento nos dois anos seguintes (65 em 2021 e 67 em 2022). Observa-se que aconteceu o inverso em comparação aos municípios classificados no parâmetro “baixo”, em que houve redução de 178 para 159 e novamente para 145. Já o parâmetro “médio” apresentou uma oscilação de zero a dois municípios no intervalo.

Com relação à distribuição espacial do indicador proporção de contatos examinados de casos novos de hanseníase diagnosticados nos anos analisados, notou-se redução do número de municípios com parâmetro “bom” no período analisado, variando de 68, em 2018, a 39, em 2022. Por outro lado, o parâmetro “precário” apresentou aumento de 29,8%, com 134 casos no início do intervalo (2018) e chegando a 174 no final (2022). O número de cidades com avaliação “regular” apresentou queda em 2018 (n=22), 2019 (n=14) e 2022 (n=7) e aumento em 2022 (n=13). Chegou a 12 municípios no último ano (Figura 3).

**Figura 3:** Distribuição espacial do indicador de proporção de contatos, referentes a novos diagnósticos, avaliados por região de desenvolvimento. Piauí, 2018-2022 (n=224)

## Discussão

A análise dos dados ao longo do quinquênio evidenciou variações nos diferentes indicadores acompanhados, com oscilações tanto no número de casos novos quanto nos demais valores observados. Identificou-se redução nos diagnósticos em 2020, ano inicial da pandemia de covid-19, seguida de aumentos nos anos subsequentes. O cálculo da prevalência oculta sugere a existência de um número ainda maior de casos, correspondentes àqueles possivelmente não detectados no período. A avaliação dos indicadores evidencia uma melhora no quadro de hiperendemicidade da doença em menores de 15 anos, mas um retrocesso na proporção de contatos examinados. Por sua vez, a proporção de novos casos com incapacidades apresenta um panorama mais incerto, devido aos efeitos da pandemia, que dificultam inferir com segurança uma tendência de melhora, agravamento ou manutenção da situação. Esses achados refletem possíveis fragilidades nas ações de vigilância e rastreamento da hanseníase.

No período de 2007 a 2021, a maior prevalência de casos da doença no estado foi registrada em Teresina, capital situada no território Entre Rios. Elevadas incidências também foram observadas nas regiões do Vale do Rio Guaribas e do Vale dos Rios Piauí e Itaueiras [14,15].

No Brasil, também entre 2018 e 2022, registraram-se 112.456 novos casos do agravo, com maior número de diagnósticos no Mato Grosso, Maranhão, Tocantins, Goiás e Piauí [4]. Os dados do Piauí revelados em nosso estudo sugerem um status alarmante, mas deve-se considerar ainda que existem falhas no processo de diagnóstico, o que pode resultar em um efeito de subnotificação [16,17]. Possíveis justificativas são desconhecimento e estigma da população, falhas de capacitação profissional e precariedade da cobertura de avaliação de contatos, o que sugere que o número real é bem maior que o identificado [18].

Além disso, o intervalo estudado sofreu impacto da pandemia de covid-19. No período, nota-se redução da taxa de detecção por 100 mil habitantes, com o menor valor atingido no ano em que se iniciou a pandemia (16,2 em 2020). Comportamento similar de redução dessa taxa, também em 2020, pode ser percebido em São Paulo e na América Latina como um todo [19,20]. No contexto pandêmico, a sobrecarga do sistema de saúde e o redirecionamento das ações para controle e combate do coronavírus, com consequente redução das atividades de diagnóstico e registro da hanseníase, podem ter contribuído para essa redução [11,21].

Entre 2001 e 2015, foram notificados mais de 240 mil casos novos de hanseníase no Nordeste, dos quais 8,7% ocorreram em crianças de 0 a 14 anos. Durante esse período, apenas os estados do Piauí, Paraíba e Bahia apresentaram declínio na detecção [22]. Nesta análise, o número de municípios com parâmetro hiperendêmico para esse indicador permaneceu em queda. No início do intervalo (2018), as regiões mostraram distribuição quase uniforme desse status. Com exceção da Planície Litorânea, todas as regiões apresentaram entre 1 e 3 municípios nessa categoria. Ao final (2022), entretanto, não foi mais observado nenhum município com essa classificação nas regiões da Planície Litorânea, Chapada das Mangabeiras, Tabuleiro do Alto Parnaíba, Tabuleiro dos Rios Piauí e Itaueiras, Vale do Canindé e Vale do Sambito.

Entre 2007 e 2021 no Piauí, constatou-se que a taxa de detecção em menores de 15 anos apresentou tendência decrescente, ainda que a média tenha permanecido em nível de hiperendemicidade nessa faixa etária [14]. Na nossa análise, observamos redução de 59% do número de municípios classificados como hiperendêmicos para a mesma taxa (de 27 para 11), paralelamente a um aumento de 7% daqueles descritos como “baixo” (de 195 para 209). Pode-se perceber redução da detecção ao longo do tempo, ainda que persistam localidades em situação hiperendêmica.

A detecção de casos nessa faixa etária (<15 anos) é um importante indicador de endemia, pois evidencia focos de transmissão ativa, em que crianças estão inseridas em ambiente de convívio com pessoas acometidas ainda sem diagnóstico ou tratamento adequado [23]. A hanseníase pode gerar impactos significativos em crianças e adolescentes, nos âmbitos físico, emocional e social, visto que os danos ocasionados pela doença podem interferir na imagem corporal e na autoestima, por meio das incapacidades físicas. Isso pode influenciar negativamente na construção da identidade e relações sociais, comprometer o desempenho escolar e até levar ao abandono dos estudos devido a discriminação [24].

O Grau de Incapacidade Física é definido a partir da Avaliação Neurológica Simplificada que determina o comprometimento neural. Os resultados podem ser: grau 0, para ausência; grau 1, para diminuição ou perda da sensibilidade; e grau 2 para presença de incapacidades e deformidades [10]. Na série histórica analisada, pode-se perceber que apenas no ano de 2022 o estado avaliou pelo menos 90,0% dos novos casos quanto à presença de incapacidades. Isso sugere um cenário de retrocesso, uma vez que, entre 2001 e 2015, o Piauí e Sergipe foram os únicos estados nordestinos a alcançarem uma média superior a esse mesmo percentual [22].

Diante disso, observa-se redução do número de pessoas com grau de incapacidade física 2 no diagnóstico até o ano de 2020. A região da Chapada das Mangabeiras destaca-se nesse comportamento, diminuindo metade o número de municípios com alta taxa de detecção (de 8 para 4). Resultado contrário é observado no cenário nacional, que apresenta aumento constante desde o ano de 2014 [4]. Apesar dessa redução local, os achados apontam para um possível cenário de falha dos serviços quanto à avaliação de incapacidades, que não é realizada em todos os pacientes no momento em que recebem o diagnóstico.

Sabe-se que o achado de Grau de Incapacidade Física 2 no diagnóstico evidencia detecção tardia. Dessa forma, esse indicador também funciona como um parâmetro indireto de endemia oculta e permite estimar uma prevalência mais realista [11,21]. Com base no cálculo de prevalência oculta no período estudado, é possível sugerir que o número de casos subnotificados cresceu durante o período, com estimativa de 1.265 ocorrências (Tabela 1). No ano de 2022, o quantitativo oculto corresponde a quase metade dos número de casos notificados (306 casos não notificados, frente aos 692 registrados). Considerando o longo período de incubação da hanseníase, essa estimativa não indica necessariamente novas infecções no ano em específico, mas casos registrados que evoluíram até o desenvolvimento de incapacidades.

Nesse sentido, o exame de contatos funciona como uma estratégia para detectar casos de forma ativa. O indicador que analisa as proporções dessas avaliações reflete a capacidade dos serviços em realizar vigilância. Considerando a importância epidemiológica dos contatos em hanseníase e do diagnóstico precoce, valores baixos podem indicar limitação de acesso aos serviços e ações de saúde, o que dificulta o alcance do controle e contribui para a endemia oculta e diagnóstico tardio [25].

Os achados deste estudo sugerem que a avaliação de contatos no período analisado apresenta uma piora constante, evidenciada pelo aumento de 29,8% do número de municípios com parâmetro “precário” para esse indicador cresceu, com crescimento absoluto em todos os anos da amostra. Esse achado difere do estimado em 2024 no estado do Amazonas. Na ocasião, evidenciou-se progressão positiva na proporção de contatos examinados no estado, variando de 73% a 88% entre 2012 e 2021 [26]. Em 2022, o cenário de precariedade desse indicador representou quase homogeneamente os agregados de cidades da região sul do estado. Ainda no mesmo ano, o Piauí ocupou a terceira posição entre os estados com maior número de municípios que atendiam a esse parâmetro, totalizando 57 cidades, atrás apenas da Bahia (106) e de Minas Gerais (78) [4].

Essa fragilidade na avaliação dos contatos pode ser atribuída ao estigma associado à hanseníase, que gera medo tanto de receber o diagnóstico quanto de comunicá-lo à família. Além disso, outros fatores, como a baixa educação em saúde, as dificuldades de acesso aos serviços e as falhas na atuação das equipes de saúde, também contribuem para esse cenário de diagnóstico tardio da doença [25].

No âmbito da Estratégia Nacional para o Enfrentamento da Hanseníase 2019-2022, o Ministério da Saúde estabeleceu metas-chave, como a redução da taxa de detecção em menores de 15 anos e da proporção de casos novos com grau 2 de incapacidade, além do combate a práticas discriminatórias contra pessoas acometidas e seus familiares, com o objetivo principal de reduzir a carga da doença no país [8]. Posteriormente, a Estratégia para o período 2024-2030 reforçou esses eixos e definiu novas metas, como a interrupção da transmissão em grande parte do território nacional, a eliminação da hanseníase em 75,0% dos municípios, a redução em 20,0% do número absoluto de casos novos com grau 2 de incapacidade no momento do diagnóstico e o atendimento integral a 100% das manifestações sobre práticas discriminatórias registradas nas Ouvidorias do SUS [9].

Neste estudo, observou-se que as taxas de detecção em menores de 15 anos permaneceram elevadas e que a proporção de casos com incapacidade no diagnóstico manteve-se expressiva, evidenciando, até o limite temporal da análise, uma dissonância com as metas estratégicas. Esses achados reforçam a necessidade de intensificação das ações previstas para o período de 2024-2030, em especial no fortalecimento da detecção oportuna, busca ativa de contatos e melhoria dos processos de notificação.

Nesse sentido, a atenção primária configura-se como protagonista no controle da hanseníase. As unidades básicas de saúde conduzem ações de vigilância e prevenção de forma descentralizada e com envolvimento da equipe multiprofissional, que deve ser capacitada para reconhecer sinais e sintomas e acompanhar os casos ativos. A busca ativa e a vigilância de contatos permitem o diagnóstico precoce, prevenindo incapacidades e complicações. Além disso, a educação em saúde integra-se às estratégias da atenção primária, favorecendo o reconhecimento da doença pela população e contribuindo para a adesão terapêutica e redução do estigma [27].

O profissional da atenção primária, em especial o enfermeiro, tem um papel indispensável na identificação de casos de hanseníase, acompanhamento, avaliação dos contatos e estímulo ao autocuidado. O combate e o controle do agravo devem ser encarados como tarefa permanente, considerando as singularidades de cada população. O investimento na vigilância epidemiológica deve ser considerado, assim como a prevenção, a busca ativa de novos casos e o acompanhamento daqueles confirmados, além do apoio dos serviços de referência encarregados do apoio matricial [3,28].

É importante destacar que este estudo apresenta limitações relacionadas à qualidade e à confiabilidade das informações, sujeitas a possíveis inconsistências no processo de notificação e registro. Por se tratar de informações secundárias, existe a possibilidade de subnotificação nos sistemas de informação, bem como limitações na interpretação da denominada prevalência oculta. Ademais, o estudo teve caráter exclusivamente descritivo, não incluindo análises estatísticas mais robustas.

 As análises foram conduzidas em nível estadual, o que limita a interpretação de variações locais, sujeitando os achados à falácia ecológica. Apesar dessas limitações, os achados oferecem uma visão abrangente da distribuição e evolução dos indicadores epidemiológicos e operacionais da hanseníase no Piauí, subsidiando ações de planejamento e controle da doença, além de estimular novos estudos focados em séries temporais e recortes regionais. 

Os valores dos indicadores analisados mostram que a hanseníase permanece classificada como hiperendêmica no estado, marcada por elevadas taxas de detecção em menores de 15 anos, alta proporção de casos com grau 2 de incapacidade e fragilidades nas ações de vigilância e controle. A grande estimativa de casos ocultos reforça a necessidade de estratégias que ampliem a capacidade de detecção precoce, fortaleçam a atenção primária e qualifiquem o processo de notificação, com o objetivo de apoiar o fomento, planejamento e realização das atividades coordenadas nas três esferas governamentais. Trata-se de algo crucial para fortalecer o controle da endemia no estado, com vistas a reduzir a transmissão e as complicações da doença.

## Data Availability

Os dados utilizados nesta pesquisa estão disponíveis em https://osf.io/pq5w3/.
